# Is atherosclerosis an autoimmune disease?

**DOI:** 10.1186/1741-7015-12-47

**Published:** 2014-03-18

**Authors:** Eiji Matsuura, Fabiola Atzeni, Piercarlo Sarzi-Puttini, Maurizio Turiel, Luis R Lopez, Michael T Nurmohamed

**Affiliations:** 1 Collaborative Research Center (OMIC), Okayama University Graduate School of Medicine, Dentistry, and Pharmaceutical Sciences, 2-5-1 Shikata-cho, Kita-ku, Okayama 700-8558, Japan; 2 Department of Cell Chemistry, Okayama University Graduate School of Medicine, Dentistry, and Pharmaceutical Sciences, Okayama, Japan; 3 Rheumatology Unit, L. Sacco University Hospital of Milan, Milan, Italy; 4 Cardiology Unit, Department of Biomedical Sciences for Health, Galeazzi Orthopedic Institute IRCCS, University of Milan, Milan, Italy; 5 Medical Department, Corgenix Inc, Broomfield, CO, USA; 6 VU University Medical Center and Jan van Breemen Research Institute, Amsterdam, Netherlands

**Keywords:** Atherosclerosis, Auto-inflammatory disease, Autoimmunity, β2-glycoprotein I, Innate immunity, Inflammasome, Oxidized LDL

## Abstract

Immunologic research into pathogenic mechanisms operating in autoimmune-mediated atherosclerosis initially focused on adaptive immunity. Current interest is directed to more basic inflammatory mechanisms. Chronic inflammation (innate immunity-associated) may trigger initial events that can lead to atherosclerotic cardiovascular disease. This chronic inflammation may start early in life and be perpetuated by classic atherosclerosis risk factors. Lipid peroxidation of low-density lipoprotein seems to be a key event in the initiation and progression of atherosclerosis. Oxidized low-density lipoprotein triggers inflammatory and immunogenic events that promote endothelial dysfunction and the synthesis and secretion of pro-inflammatory cytokines, leading to an autoimmune response capable of accelerating the intracellular accumulation of lipids within atherosclerotic plaques. Oxidized low-density lipoprotein binds β2-glycoprotein I to form circulating complexes found in both autoimmune and non-autoimmune atherosclerosis. It is likely that β2-glycoprotein I and/or these complexes contribute to early atherogenesis by stimulating pro-inflammatory innate immunity through endogenous sensors and inflammasome/interleukin-1 pathways. We discuss the chronic inflammatory (innate) and autoimmune (adaptive) responses operating in atherosclerosis to discern the role of autoimmunity in atherosclerotic cardiovascular disease.

## Background

In the last decades, it has become apparent that patients with systemic autoimmune diseases develop premature and, quite often, severe atherosclerotic cardiovascular disease (CVD). Systemic autoimmune diseases are characterized by chronic inflammation and immune dysregulation. These abnormalities may produce dyslipidemia, platelet and vascular pathology, arterial lesions, and enhanced autoantibody production [1-3]. Current narrative for non-autoimmune atherosclerosis emphasizes the active inflammatory, complex or multi-factorial and long-term nature of the disease. These inflammatory mechanisms also cause dyslipidemia with vascular and immunologic dysfunction. Giving the similarities with autoimmune-mediated atherosclerosis, it is not surprising that investigators have postulated an autoimmune nature for atherosclerosis.

Innate immune mechanisms have been demonstrated in atherosclerosis, particularly in early stages of the disease. Unraveling the roles of inflammatory molecular factors and signaling systems activated by a variety of pathogens and/or endogenous signals highlighted the prominent role of pro-inflammatory inflammasome/IL-1 cytokines and turned attention to auto-inflammatory mechanisms in atherosclerosis [4,5]. Whether these pro-inflammatory mechanisms progress into atherogenic adaptive immune responses in late stages of the disease or represent two independent pathologic processes remains unresolved. Understanding their nature and inter-relationship in atherosclerosis may provide new concepts with possible impact not only on early and accurate diagnosis but also on preventive programs and perhaps more effective therapeutic interventions.

### Chronic inflammatory mechanisms and immune dysregulation in atherosclerosis

There is much evidence to suggest that endothelial dysfunction (the *primum movens*) is an early pro-atherogenic process associated with cardiovascular events that contributes to the formation, progression and complications of atherosclerosis [6]. It is also becoming increasingly clear that chronic inflammation and immune dysregulation play important roles in the development of atherosclerotic CVD, which can now be considered an inflammatory autoimmune condition [6,7].

Oxidized low-density lipoprotein (oxLDL) is pivotal in the development of atherosclerosis and represents a crucial pro-inflammatory stimulus [8]. Upon entering into the intima of arteries, oxLDL activates endothelial cells and up-regulates adhesion molecule expression and chemotactic chemokine secretion, all of which contribute to the recruitment of circulating leukocytes. Monocytes and/or macrophages infiltrating atherosclerotic sites take up oxLDL, forming ‘foam cells’ that in turn promote further secretion of inflammatory mediators. The association between oxLDL autoantibodies and CVD in patients with rheumatoid arthritis (RA) has been reported [8]. OxLDL may interact with C-reactive protein (CRP) to form pro-atherogenic oxLDL/CRP complexes that may not only perpetuate vascular inflammation but also trigger autoimmune responses, accelerating the development of atherosclerosis [9]. OxLDL/β2-glycoprotein I (β2GPI) complexes also induce adaptive autoimmune responses, which may up-regulate the macrophage expression of FcγRI and scavenger CD36 receptors and thus accelerate oxLDL uptake. OxLDL/β2GPI complexes correlate with the size of atherosclerotic lesions in mouse models. In patients with CVD, these complexes correlate with disease severity and adverse outcomes [5].

Chronic inflammation is a major component of atherogenesis, and both *in vitro* and *in vivo* studies have shown that IL-1β is a potent pro-inflammatory and atherogenic cytokine [10,11]. The production of IL-1β depends on two separate signals: the induction of IL-1β mRNA as a result of the stimulation of pattern recognition receptors; and the activation of caspase-1, a protease that cleaves pro-IL-1β into its biologically active form [12]. The activation of caspase-1 is mediated by cytoplasmic large multi-protein complexes, called inflammasomes, that cleave pro-caspase-1 into its mature activated proteinase form. Caspase-1 is a key bridge linking metabolic stresses and innate immune sensors to produce pro-inflammatory cytokines and vascular inflammation [13].

IL-1 is a potent pro-inflammatory cytokine with a wide range of biological effects. IL-1β can be induced during infection, metabolic or endogenous injury [14], or immunological challenge and can control systemic and local inflammation by up-regulating the expression of many effector proteins through the stimulation of IL-1 receptors and the NF-κB pathway. It also stimulates the synthesis of IL-6, fibrinogen, CRP and the other inflammatory mediators involved in coronary artery syndromes. Furthermore, increased IL-1β mRNA levels in human atherosclerotic plaques suggests that locally synthesized IL-1 protein may activate the synthesis of growth factors and other cytokines, leading to local inflammatory cascades [12].

Various studies have shown that Toll-like receptor (TLR) signaling is operative in the intima of atherosclerotic arteries, with a number of pathogens and endogenous TLR ligands in atherosclerotic lesions. It is therefore conceivable that, together with other pro-atherogenic ligands, cholesterol crystals can elicit inflammasome/IL-1 inflammatory responses [15], a hypothesis supported by the finding that caspase-1 mRNA and caspase protein are present in atherosclerotic lesions but not in normal arteries. Cholesterol crystals can induce inflammasome nucleotide-binding domain leucine-rich repeat receptor protein 3 (NLRP3) activation with IL-1β and IL-18 production. Activated caspase-1 has been observed in ruptured but not stable plaque in patients experiencing sudden coronary death, and patients with high plasma caspase-1 levels have significantly lower survival rates after a myocardial infarction [13]. As inflammasome-mediated IL-1β release promotes an inflammatory milieu and drives lesion progression; cholesterol crystal-induced inflammasome activation may be an important link between cholesterol metabolism and inflammation in atherosclerotic lesions.

Atherosclerosis should therefore be considered an auto-inflammatory (not autoimmune) disease that triggers the production of autoantibodies against substances such as oxLDL [16].

### The role of β2GPI in atherogenesis

Dysregulation of inflammatory responses and adaptive immunity are important pathologic mechanisms underlying the clinical manifestations of systemic lupus erythematosus (SLE) and antiphospholipid syndrome. These patients may develop venous thromboembolism and premature CVD.

The emerging role of anti-β2GPI and anti-oxLDL antibodies in autoimmune-mediated atherothrombosis was first recognized in patients with antiphospholipid syndrome [17]. The systemic and local generation of free radicals by inflammatory cells may induce oxidative modifications of LDL. OxLDL generation and uptake by arterial mononuclear cells activate inflammatory and chemotactic cytokines in early stages of atherosclerosis, followed by an excessive oxLDL intracellular accumulation [18]. β2GPI was initially described as a natural anticoagulant, but it has more pleiotropic functions affecting fibrinolysis, angiogenesis, apoptosis and atherogenesis [19]. OxLDL binds β2GPI via specific oxidized lipid epitopes forming pro-atherogenic oxLDL/β2GPI complexes [20]. This interaction suggests an anti-oxidant role of β2GPI by quenching the pro-inflammatory and pro-atherogenic effects of oxLDL. OxLDL/β2GPI complexes may also become immunogenic, triggering the production of autoantibodies. Current evidence indicates the atherosclerotic lesion as the primary site for oxLDL/β2GPI complex formation. Because oxLDL/β2GPI and their immune complexes up-regulate the expression of scavenger and FcγRI receptors, with rapid accumulation in lysosomes where danger signals are processed, oxLDL and β2GPI may also contribute to the activation of inflammasomes [5].

Innate immunity provides a first line of host defense against a variety of pathogens and endogenous danger signals. Cells of the innate immune system mediate inflammatory responses through signaling receptors to detect pathogens or altered endogenous molecules [21]. These receptors recognize pathogen-associated molecular patterns including damaged tissue and modified self-molecules [10]. TLRs are extracellular pathogen-associated molecular pattern sensors capable of triggering signaling cascades that lead to the expression of pro-inflammatory cytokines, such as tumor necrosis factor alpha and IL-1 via the NF-κB pathway [22]. There are two families of intracellular receptors: retinoic acid-inducible gene I-like helicases and NLRs [23,24]. In particular, NLRP3 can activate pro-inflammatory caspases in response to endogenous stimuli. Activated caspase-1 controls the maturation of the pro-inflammatory IL-1 family of cytokines. Inflammasomes are multi-molecular complexes of NLRP3 that regulate inflammatory caspases and IL-1 production. IL-1β is a potent pro-inflammatory cytokine with deleterious effects if produced uncontrollably.

Intracellular NLRP3 can be assembled by lysosomal damage and reactive oxygen species. Thus, host-derived oxidation-specific epitopes may activate innate immunity through a variety of receptors for danger (or damage)-associated molecular patterns [25]. Lipid peroxidation is ubiquitous and represents a major component of an atherosclerotic inflammatory state. Endogenous atherogenic danger signals such as oxLDL and oxLDL/β2GPI may be capable of dysregulating an inflammasome-induced IL-1β response, representing an early inflammatory mechanism in atherogenesis.

High circulating levels of oxLDL/β2GPI may indicate an advanced endogenous process that is already disseminated (systemic) and their effects can be detectable by other means, such as intima media thickness (IMT), angiography or imaging. Further, if an antibody response to any of these elements is present (that is, anti-oxLDL, anti-β2GPI or anti-oxLDL/β2GPI), the process may have already reached the level of an adaptive immune response causing a full clinical disease expression, possibly indicating high risk for adverse outcomes. The measurement of these biomarkers may provide specific information about an underlying atherogenic inflammasome/IL-1β response. Intervention (prevention) may be more effective at this level as has been shown with classical auto-inflammatory diseases.

### Pro-atherothrombotic milieu in systemic autoimmunity

Nowadays, atherosclerosis is seen as an inflammatory disease of the arterial vessel [26], and it is therefore not surprising that the chronic inflammatory disorder RA is associated with an increased cardiovascular risk. But autoimmunity also plays a role, because patients with RA who are rheumatoid negative or anti-cyclic citrullinated peptide negative have less atherosclerotic disease than patients with RA who have these autoantibodies. Moreover, SLE is also associated with an increased cardiovascular risk.

Cells of both the innate and adaptive immune systems are involved in atherogenesis. The importance of adaptive immunity was demonstrated in experimental models such as apolipoprotein E-deficient mice. T and B cell deficiency reduces the atherosclerotic burden by 40% to 80%, in both early and late phases of atherosclerotic disease [27,28]. Most T cells in atherosclerotic plaques are CD4+ and express the αβ-T-cell receptor, which interacts with major histocompatibility complex class II molecules. T cells infiltrating atherosclerosis lesions have a Th1 profile that activate macrophages and increases pro-inflammatory cytokines such as IFN-γ. IFN-γ decreases collagen synthesis, making the atherosclerotic fibrous cap more vulnerable to rupture or thrombosis. Th17 lymphocytes represent another T helper subset involved in inflammation but their precise role in atherosclerosis is still under investigation. The protective role of regulatory T cells in atherosclerosis was demonstrated in patients with acute coronary syndrome who had low regulatory T cell levels compared with patients with stable angina and normal coronary arteries [29,30]. A pro-inflammatory subset of CD4+ T cells lacking the co-stimulatory receptor CD28 (CD4+, CD28-) is increased in atherosclerotic plaques from patients with unstable angina [31,32].

Antiphospholipid and anti-oxLDL antibodies cross-react and anti-oxLDL/β2GPI complexes are increased in SLE and RA, providing an initial explanation for the increased cardiovascular risk in autoimmunity. In a population-based cohort, the presence of antinuclear antibodies (ANA) correlated with cardiovascular events and mortality [33]. The presence of ANA was a significant independent predictor of cardiovascular events and death, with hazards ratios of 1.26 and 1.18, respectively. The role of ANA in preclinical atherosclerosis was investigated in The Cardiovascular Risk in Young Finns Study [34] of 2,278 participants. ANA positivity was observed in 10.5% of women and 4.5% of men. Multivariate analyses, adjusted for age, body mass index, CRP, lipids, blood pressure and smoking habits, showed that ANA positivity was inversely associated with carotid compliance in women. ANA might thus have a role in the development of early atherosclerosis through induction of endothelial dysfunction.

TLR signaling by innate immune cells is also important for the development of atherosclerotic lesions [35]. TLRs are also essential for the adaptive immune system because they activate dendritic cells and macrophages with subsequent T and B cell activation. Important endogenous TLR ligands in RA are gp 96 and tenascin C. The synovial expression of gp 96 increases with inflammation, which activates macrophages through TLR2 and TLR4. Inflammation increases the expression of tenascin-C, activating TLR4. Other TLR ligands include serum amyloid A, heat shock proteins and high mobility group box chromosomal protein 1.

Autoimmune disease and atherosclerosis have a number of pathogenic similarities [36]. Chronic inflammation underlies both diseases with increased numbers of macrophages, dendritic cells, and B and T lymphocytes. TLR signaling is also essential in both diseases; however, the endogenous ligands differ, for example, lipids do not function as endogenous ligands in RA synovial tissue. However, gp96, tenascin C or high-mobility group box 1 protein released from the arthritic joint might function as ligands for macrophages in the atherosclerotic plaque, with subsequent progression of the lesion. In SLE, RNA and DNA antibodies can either directly or indirectly (through plasmacytoid dendritic cells) lead to increased plaque inflammation and rupture as the ultimate consequence.

## Conclusions

There is increasing evidence that autoimmunity plays an essential role in atherogenesis. TLR and cytoplasmic (inflammasome NLRP3) receptors, with their signaling pathways, are essential components of both the innate and adaptive immune systems that participate in the development of autoimmune diseases as well as in atherosclerosis. Systemic autoimmune diseases and atherosclerosis share common pathogenic pathways. A chronic inflammatory background mediated by the TLR and inflammasome/IL-1 pathways as seen in auto-inflammatory diseases and endothelial dysfunction are early steps that may lead to the development of atherosclerotic plaques. Several human studies of auto-inflammatory disease have indicated that this stage may still be reversible. It is tempting to speculate that autoantibodies (an essential component of autoimmune diseases) may ultimately cause structural and irreversible arterial wall damage with subsequent atherosclerotic plaque development and rupture.

OxLDL and β2GPI are both inflammatory (innate) and immunogenic (adaptive) molecules. One possible role for these molecules is that they may serve as biological links bridging the progression from chronic inflammation into a full autoantibody response in later stages of atherosclerosis. When autoantibodies are present, patients with RA have an accelerated atherosclerosis in comparison to patients with RA who do not have these antibodies. Similarly, autoantibodies in SLE amplify atherosclerosis. Thus, whether atherosclerosis is an auto-inflammatory disease, an autoimmune disease, or both is still debatable, with sound arguments supporting each position.

## Abbreviations

ANA: antinuclear antibodies; CRP: C-reactive proteins; CVD: cardiovascular disease; IFN-γ: interferon gamma; IL: interleukin; NF-κB: nuclear factor kappa B; NLR: nucleotide-binding domain leucine-rich repeat receptor; oxLDL: oxidized low-density lipoproteins; RA: rheumatoid arthritis; SLE: systemic lupus erythematosus; TLR: Toll-like receptor; β2GPI: β2-glycoprotein I.

## Competing interests

The authors declare that they have no competing interest.

## Authors’ contributions

All authors contributed equally. All authors read and approved the final manuscript.
